# (*E*)-2-{2-*tert*-Butyl-6-[2-(4-hy­droxy­phen­yl)ethen­yl]-1-propyl-1,4-dihydro­pyridin-4-yl­idene}indane-1,3-dione

**DOI:** 10.1107/S1600536810051044

**Published:** 2010-12-15

**Authors:** Kwang Ha, Sae Byul Park, Hyung Jin Kim

**Affiliations:** a School of Applied Chemical Engineering, Chonnam National University, Gwangju 500-757, Republic of Korea

## Abstract

The title compound, C_29_H_29_NO_3_, the nearly planar nine-membered indanedione ring [maximum deviation = 0.027 (2) Å] is located approximately parallel to its carrier pyridine ring [maximum deviation = 0.021 (2) Å] with a dihedral angle of 1.8 (1)° between the planes. However, because of steric hindrance, the benzene ring [maximum deviation = 0.006 (2) Å] is not parallel to the pyridine ring [dihedral angle = 37.29 (8)°]. The mol­ecules display numerous inter­molecular π–π inter­actions between the five- and six-membered rings, the shortest centroid–centroid distance being 3.796 (2) Å. There are inter- and intra­molecular O—H⋯O and C—H⋯O hydrogen bonds.

## Related literature

For the synthesis of the starting material, see: Yao *et al.* (2006*a* ▶). For the synthesis of the title compound, see: Peng *et al.* (2006 ▶). For background to luminescent materials, see: Andreu *et al.* (2009 ▶); Kim *et al.* (2004 ▶); Yao *et al.* (2006*a* ▶,*b* ▶).
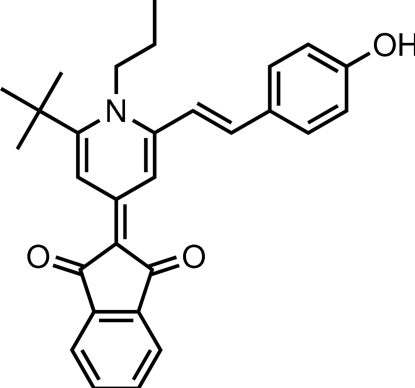


## Experimental

### 

#### Crystal data

C_29_H_29_NO_3_
*M* *_r_* = 439.53
Monoclinic, 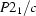

*a* = 9.1330 (8) Å
*b* = 12.4857 (12) Å
*c* = 20.440 (2) Å
β = 97.775 (3)°
*V* = 2309.4 (4) Å^3^
*Z* = 4
Mo *K*α radiation
μ = 0.08 mm^−1^
*T* = 200 K
0.25 × 0.15 × 0.10 mm

#### Data collection

Bruker SMART 1000 CCD diffractometer
Absorption correction: multi-scan (*SADABS*; Bruker, 2000 ▶) *T*_min_ = 0.809, *T*_max_ = 1.000
16959 measured reflections
5724 independent reflections
2458 reflections with *I* > 2σ(*I*)
*R*_int_ = 0.097

#### Refinement

*R*[*F* ^2^ > 2σ(*F* ^2^)] = 0.063
*wR*(*F* ^2^) = 0.144
*S* = 0.95
5724 reflections
303 parameters
H-atom parameters constrained
Δρ_max_ = 0.27 e Å^−3^
Δρ_min_ = −0.24 e Å^−3^

### 

Data collection: *SMART* (Bruker, 2000 ▶); cell refinement: *SAINT* (Bruker, 2000 ▶); data reduction: *SAINT*; program(s) used to solve structure: *SHELXS97* (Sheldrick, 2008 ▶); program(s) used to refine structure: *SHELXL97* (Sheldrick, 2008 ▶); molecular graphics: *ORTEP-3* (Farrugia, 1997 ▶) and *PLATON* (Spek, 2009 ▶); software used to prepare material for publication: *SHELXL97*.

## Supplementary Material

Crystal structure: contains datablocks global, I. DOI: 10.1107/S1600536810051044/pb2048sup1.cif

Structure factors: contains datablocks I. DOI: 10.1107/S1600536810051044/pb2048Isup2.hkl

Additional supplementary materials:  crystallographic information; 3D view; checkCIF report

## Figures and Tables

**Table 1:** Hydrogen-bond geometry (Å, °)

*D*—H⋯*A*	*D*—H	H⋯*A*	*D*⋯*A*	*D*—H⋯*A*
O1—H1⋯O3^i^	0.84	1.79	2.626 (2)	177
C2—H2⋯O2	0.95	2.24	2.927 (3)	128
C4—H4⋯O3	0.95	2.29	2.966 (3)	127
C15—H15⋯O3^i^	0.95	2.55	3.210 (3)	127
C16—H16⋯O1^ii^	0.95	2.57	3.500 (3)	166
C29—H29*B*⋯O2^iii^	0.98	2.59	3.515 (3)	158

Symmetry codes: (i) 
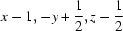
; (ii) 
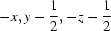
; (iii) 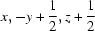.

**Fig. 1.**
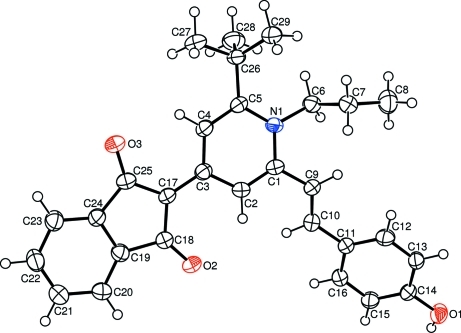
The structure of the title compound, with displacement ellipsoids drawn at the 50% probability level for non-H atoms.

**Fig. 2.**
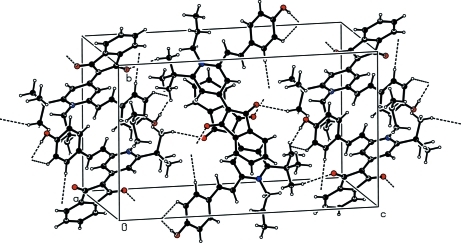
View of the unit-cell contents of the title compound. Hydrogen-bond interactions are drawn with dashed lines.

## supplementary crystallographic information

### Comment

Indene-1,3(2*H*)-dione moiety is used as a strong electron acceptor for luminescent materials such as organic light-emitting diodes (OLED) (Yao *et al.*, 2006*a*,*b*; Andreu *et al.*, 2009; Kim *et al.*, 2004). For the purpose of finding a pH-sensing luminescent dye, we designed the title compound which includes indene-1,3(2*H*)-dione moiety conjugated with 1,4-dihydropyridine ring possessing a 4-hydroxystyryl group. The title compound was synthesized by the Knoevenagel condensation of 2-(2-*tert*-butyl-6-methyl-1-propylpyridin-4(1*H*)-ylidene)-1*H*-indene-1,3(2*H*)-dione with 4-hydroxybenzaldehyde in a sealed tube and its structure was confirmed by NMR spectra and X-ray crystal analysis.

The title compound, C_29_H_29_NO_3_, is a 1,4-dihydropyridine ring with four distinct substituents (Fig. 1). In the crystal structure, the nearly planar 9-membered ring [maximum deviation of 0.027 (2) Å for C17] is located approximately parallel to its carrier pyridine ring [maximum deviation of 0.021 (2) Å for C3] with the dihedral angle of 1.8 (1)° between the planes. However, because of the steric hindrance, the benzne ring [maximum deviation of 0.006 (2) Å for C14] is not parallel to the pyridine ring. The dihedral angle between the pyridine and the benzene rings is 37.29 (8)°. The molecules display numerous intermolecular π-π interactions between the 5- and 6-membered rings. The shortest centroid-centroid distance is 3.796 (2) Å and the dihedral angle between the ring planes is 1.7 (1)°. Moreover, there are inter- and intramolecular O—H···O and C—H···O hydrogen bonds with d(O···O) = 2.626 (2) Å and d(C···O) = 2.927 (3) Å–3.515 (3) Å (Fig. 2, Table 1).

### Experimental

A mixture of 2-(2-*tert*-butyl-6-methyl-4*H*-pyran-4-ylidene)-1*H*-indene-1,3(2*H*)-dione (900 mg, 7.13 mmol) and propylamine (15 ml) was heated at 150 °C for 3 h. The mixture was cooled and concentrated under vacuum. The residue was crystallized from CHCl_3_ to give 2-(2-*tert*-butyl-6-methyl-1-propylpyridin-4(1*H*)-ylidene)-1*H*-indene-1,3(2*H*)-dione (750 mg, 73%). A mixture of 2-(2-*tert*-butyl-6-methyl-1-propylpyridin-4(1*H*)-ylidene)-1*H*-indene-1,3(2*H*)-dione (500 mg, 1.5 mmol), 4-hydroxybenzaldehyde (364 mg, 3.0 mmol), piperidine (600 mg, 7.0 mmol), n-butanol (10 ml) and molecular sieve (4 Å, 3 g) was heated in a sealed tube at 140 °C for 12 h. The reaction mixture was filtered and the filtrate was concentrated under vacuum to give crude product, which was chromatographed on SiO_2_ eluting with a mixture of CHCl_3_/acetone (10:1) solution to afford the title compound (130 mg, 20%) as a yellow solid. Crystals suitable for X-ray analysis were obtained by slow evaporation from a CHCl_3_/EtOH solution at room temperature. Mp 211 °C (dec.). ^1^H NMR (300 MHz, DMSO-d_6_): δ 9.94 (s, 1H, O*H*), 8.91 (d, 1H, J = 1.85 Hz, C—C*H*=C—N), 8.81 (d, 1H, J = 1.85 Hz, C—C*H*=C—N), 7.62–6.84 (m, 10H, Ar), 7.42 (d, 1H, J = 15.6 Hz, *H*C=CH—C_6_H_4_OH), 7.22 (d, 1H, J = 15.6 Hz, HC=C*H*-C_6_H_4_OH), 4.48 (t, 2H, J = 8.1 Hz, NC*H*_2_CH_2_CH_3_), 2.0 (m, 2H, NCH_2_C*H*_2_CH_3_), 1.53 [s, 9H, –C(C*H*_3_)_3_], 0.88 (t, 3H, J = 7.2 Hz, NCH_2_CH_2_C*H*_3_). ^13^C NMR (75 MHz, DMSO-d_6_): δ 190.5, 158.6, 158.5, 151.9, 148.4, 139.3, 137.5, 131.2, 128.9, 126.1, 118.8, 117.8, 118.0, 115.2, 113.1, 112.9, 51.1, 36.7, 30.2, 22.9, 9.8.

### Refinement

H atoms were positioned geometrically and allowed to ride on their respective parent atoms [C—H = 0.95 (CH), 0.99 (CH_2_) or 0.98 Å (CH_3_) and O—H = 0.84 Å, and *U*_iso_(H) = 1.2*U*_eq_(C) or 1.5*U*_eq_(methyl C, O)].

### Crystal data

**Table:**

C_29_H_29_NO_3_	*F*(000) = 936
*M**_r_* = 439.53	*D*_x_ = 1.264 Mg m^−^^3^
Monoclinic, *P*2_1_/*c*	Mo *K*α radiation, λ = 0.71073 Å
Hall symbol: -P 2ybc	Cell parameters from 1849 reflections
*a* = 9.1330 (8) Å	θ = 2.6–22.5°
*b* = 12.4857 (12) Å	µ = 0.08 mm^−^^1^
*c* = 20.440 (2) Å	*T* = 200 K
β = 97.775 (3)°	Block, orange
*V* = 2309.4 (4) Å^3^	0.25 × 0.15 × 0.10 mm
*Z* = 4	

### Data collection

**Table:**

Bruker SMART 1000 CCD diffractometer	5724 independent reflections
Radiation source: fine-focus sealed tube	2458 reflections with *I* > 2σ(*I*)
graphite	*R*_int_ = 0.097
φ and ω scans	θ_max_ = 28.3°, θ_min_ = 1.9°
Absorption correction: multi-scan (*SADABS*; Bruker, 2000)	*h* = −12→9
*T*_min_ = 0.809, *T*_max_ = 1.000	*k* = −14→16
16959 measured reflections	*l* = −27→27

### Refinement

**Table:**

Refinement on *F*^2^	Primary atom site location: structure-invariant direct methods
Least-squares matrix: full	Secondary atom site location: difference Fourier map
*R*[*F*^2^ > 2σ(*F*^2^)] = 0.063	Hydrogen site location: inferred from neighbouring sites
*wR*(*F*^2^) = 0.144	H-atom parameters constrained
*S* = 0.95	*w* = 1/[σ^2^(*F*_o_^2^) + (0.0389*P*)^2^] where *P* = (*F*_o_^2^ + 2*F*_c_^2^)/3
5724 reflections	(Δ/σ)_max_ < 0.001
303 parameters	Δρ_max_ = 0.27 e Å^−^^3^
0 restraints	Δρ_min_ = −0.24 e Å^−^^3^

### Special details

**Table:**

Geometry. All e.s.d.'s (except the e.s.d. in the dihedral angle between two l.s. planes) are estimated using the full covariance matrix. The cell e.s.d.'s are taken into account individually in the estimation of e.s.d.'s in distances, angles and torsion angles; correlations between e.s.d.'s in cell parameters are only used when they are defined by crystal symmetry. An approximate (isotropic) treatment of cell e.s.d.'s is used for estimating e.s.d.'s involving l.s. planes.
Refinement. Refinement of *F*^2^ against ALL reflections. The weighted *R*-factor *wR* and goodness of fit *S* are based on *F*^2^, conventional *R*-factors *R* are based on *F*, with *F* set to zero for negative *F*^2^. The threshold expression of *F*^2^ > σ(*F*^2^) is used only for calculating *R*-factors(gt) *etc*. and is not relevant to the choice of reflections for refinement. *R*-factors based on *F*^2^ are statistically about twice as large as those based on *F*, and *R*- factors based on ALL data will be even larger.

### Fractional atomic coordinates and isotropic or equivalent isotropic displacement parameters (Å^2^)

**Table:**

	*x*	*y*	*z*	*U*_iso_*/*U*_eq_	
O1	−0.0695 (2)	0.59992 (15)	−0.28744 (9)	0.0476 (5)	
H1	−0.1176	0.5587	−0.3149	0.071*	
O2	0.5781 (2)	0.11427 (16)	−0.09052 (9)	0.0486 (6)	
O3	0.78458 (19)	0.03498 (15)	0.12927 (8)	0.0457 (5)	
N1	0.4052 (2)	0.36029 (17)	0.09620 (10)	0.0321 (5)	
C1	0.3979 (3)	0.3394 (2)	0.02930 (12)	0.0341 (7)	
C2	0.4809 (3)	0.2590 (2)	0.00776 (12)	0.0358 (7)	
H2	0.4768	0.2474	−0.0384	0.043*	
C3	0.5722 (3)	0.1927 (2)	0.05153 (12)	0.0321 (6)	
C4	0.5788 (3)	0.2198 (2)	0.11904 (12)	0.0337 (7)	
H4	0.6430	0.1795	0.1502	0.040*	
C5	0.4981 (3)	0.3010 (2)	0.14242 (12)	0.0301 (6)	
C6	0.3013 (3)	0.4443 (2)	0.11389 (12)	0.0372 (7)	
H6A	0.2879	0.4352	0.1608	0.045*	
H6B	0.2039	0.4332	0.0870	0.045*	
C7	0.3518 (3)	0.5595 (2)	0.10358 (13)	0.0395 (7)	
H7A	0.4173	0.5838	0.1434	0.047*	
H7B	0.4087	0.5618	0.0657	0.047*	
C8	0.2198 (3)	0.6337 (2)	0.09059 (15)	0.0544 (9)	
H8A	0.1540	0.6087	0.0517	0.082*	
H8B	0.2537	0.7064	0.0826	0.082*	
H8C	0.1662	0.6339	0.1290	0.082*	
C9	0.3034 (3)	0.4045 (2)	−0.01866 (12)	0.0375 (7)	
H9	0.2809	0.4756	−0.0068	0.045*	
C10	0.2472 (3)	0.3688 (2)	−0.07848 (13)	0.0372 (7)	
H10	0.2638	0.2954	−0.0874	0.045*	
C11	0.1628 (3)	0.4304 (2)	−0.13161 (12)	0.0317 (6)	
C12	0.1454 (3)	0.5414 (2)	−0.12959 (13)	0.0390 (7)	
H12	0.1889	0.5799	−0.0918	0.047*	
C13	0.0667 (3)	0.5963 (2)	−0.18120 (12)	0.0364 (7)	
H13	0.0554	0.6717	−0.1785	0.044*	
C14	0.0037 (3)	0.5414 (2)	−0.23731 (12)	0.0336 (7)	
C15	0.0185 (3)	0.4315 (2)	−0.24032 (13)	0.0382 (7)	
H15	−0.0254	0.3933	−0.2782	0.046*	
C16	0.0974 (3)	0.3770 (2)	−0.18809 (12)	0.0363 (7)	
H16	0.1073	0.3015	−0.1907	0.044*	
C17	0.6529 (3)	0.1067 (2)	0.02824 (12)	0.0323 (6)	
C18	0.6471 (3)	0.0741 (2)	−0.04036 (13)	0.0351 (7)	
C19	0.7476 (3)	−0.0205 (2)	−0.04171 (13)	0.0350 (7)	
C20	0.7841 (3)	−0.0786 (2)	−0.09424 (13)	0.0430 (7)	
H20	0.7412	−0.0628	−0.1381	0.052*	
C21	0.8859 (3)	−0.1616 (2)	−0.08116 (14)	0.0467 (8)	
H21	0.9122	−0.2034	−0.1166	0.056*	
C22	0.9486 (3)	−0.1837 (2)	−0.01795 (15)	0.0463 (8)	
H22	1.0183	−0.2403	−0.0103	0.056*	
C23	0.9122 (3)	−0.1248 (2)	0.03534 (14)	0.0414 (7)	
H23	0.9564	−0.1403	0.0791	0.050*	
C24	0.8103 (3)	−0.0435 (2)	0.02303 (13)	0.0344 (7)	
C25	0.7502 (3)	0.0347 (2)	0.06786 (13)	0.0354 (7)	
C26	0.5070 (3)	0.3201 (2)	0.21726 (12)	0.0340 (7)	
C27	0.6370 (3)	0.2581 (2)	0.25452 (12)	0.0469 (8)	
H27A	0.6237	0.1814	0.2456	0.070*	
H27B	0.6413	0.2712	0.3020	0.070*	
H27C	0.7293	0.2822	0.2398	0.070*	
C28	0.3691 (3)	0.2742 (2)	0.24283 (13)	0.0512 (8)	
H28A	0.2806	0.3096	0.2203	0.077*	
H28B	0.3759	0.2867	0.2905	0.077*	
H28C	0.3629	0.1970	0.2341	0.077*	
C29	0.5324 (3)	0.4375 (2)	0.23891 (13)	0.0455 (8)	
H29A	0.6095	0.4689	0.2159	0.068*	
H29B	0.5635	0.4404	0.2867	0.068*	
H29C	0.4404	0.4779	0.2279	0.068*	

### Atomic displacement parameters (Å^2^)

**Table:**

	*U*^11^	*U*^22^	*U*^33^	*U*^12^	*U*^13^	*U*^23^
O1	0.0611 (14)	0.0377 (13)	0.0384 (12)	0.0059 (10)	−0.0140 (10)	0.0000 (10)
O2	0.0662 (14)	0.0522 (14)	0.0251 (11)	0.0169 (11)	−0.0023 (9)	0.0015 (10)
O3	0.0559 (12)	0.0513 (14)	0.0271 (11)	0.0104 (10)	−0.0050 (9)	−0.0016 (10)
N1	0.0349 (13)	0.0362 (14)	0.0256 (12)	0.0032 (11)	0.0055 (10)	−0.0029 (11)
C1	0.0396 (17)	0.0377 (18)	0.0243 (15)	0.0012 (14)	0.0024 (12)	0.0001 (13)
C2	0.0439 (17)	0.0381 (18)	0.0251 (15)	0.0048 (14)	0.0038 (13)	−0.0022 (13)
C3	0.0356 (16)	0.0319 (17)	0.0284 (15)	−0.0024 (13)	0.0030 (12)	0.0017 (13)
C4	0.0385 (16)	0.0341 (17)	0.0270 (15)	0.0018 (14)	−0.0009 (12)	0.0026 (13)
C5	0.0327 (15)	0.0312 (16)	0.0256 (15)	−0.0038 (13)	0.0011 (12)	0.0031 (12)
C6	0.0384 (16)	0.0415 (19)	0.0326 (16)	0.0056 (14)	0.0077 (12)	−0.0002 (14)
C7	0.0438 (17)	0.0395 (18)	0.0355 (17)	0.0047 (15)	0.0067 (13)	−0.0020 (14)
C8	0.055 (2)	0.046 (2)	0.062 (2)	0.0085 (17)	0.0029 (16)	0.0025 (17)
C9	0.0462 (17)	0.0383 (18)	0.0274 (16)	0.0111 (14)	0.0030 (13)	0.0014 (14)
C10	0.0419 (17)	0.0333 (17)	0.0363 (17)	0.0051 (14)	0.0044 (13)	0.0029 (14)
C11	0.0343 (16)	0.0318 (17)	0.0287 (15)	0.0019 (13)	0.0037 (12)	−0.0002 (13)
C12	0.0439 (17)	0.0410 (19)	0.0305 (16)	−0.0022 (15)	−0.0005 (13)	−0.0043 (14)
C13	0.0447 (17)	0.0290 (16)	0.0340 (17)	0.0035 (14)	−0.0004 (13)	−0.0007 (13)
C14	0.0344 (16)	0.0354 (18)	0.0297 (16)	0.0026 (14)	0.0002 (12)	0.0034 (14)
C15	0.0439 (18)	0.0381 (18)	0.0306 (16)	−0.0016 (14)	−0.0028 (13)	−0.0035 (14)
C16	0.0404 (17)	0.0321 (17)	0.0360 (17)	0.0050 (14)	0.0037 (13)	−0.0011 (13)
C17	0.0363 (16)	0.0350 (17)	0.0242 (15)	0.0025 (13)	−0.0006 (12)	0.0011 (12)
C18	0.0390 (17)	0.0353 (17)	0.0305 (16)	0.0034 (14)	0.0034 (13)	−0.0003 (13)
C19	0.0369 (16)	0.0330 (17)	0.0345 (16)	0.0003 (14)	0.0034 (12)	−0.0038 (13)
C20	0.0517 (19)	0.045 (2)	0.0311 (16)	0.0075 (16)	0.0018 (13)	−0.0045 (14)
C21	0.051 (2)	0.046 (2)	0.044 (2)	0.0085 (16)	0.0124 (15)	−0.0026 (16)
C22	0.0456 (19)	0.0389 (19)	0.054 (2)	0.0112 (15)	0.0057 (15)	−0.0035 (16)
C23	0.0417 (18)	0.0390 (19)	0.0414 (18)	0.0041 (15)	−0.0020 (14)	−0.0007 (15)
C24	0.0377 (16)	0.0333 (17)	0.0308 (16)	0.0029 (14)	−0.0004 (12)	0.0006 (13)
C25	0.0376 (17)	0.0404 (18)	0.0269 (16)	−0.0019 (14)	0.0002 (12)	−0.0013 (14)
C26	0.0436 (17)	0.0360 (17)	0.0225 (15)	−0.0013 (14)	0.0046 (12)	−0.0016 (13)
C27	0.061 (2)	0.054 (2)	0.0254 (16)	0.0025 (16)	0.0018 (14)	0.0006 (14)
C28	0.060 (2)	0.057 (2)	0.0398 (18)	−0.0049 (17)	0.0157 (15)	0.0014 (16)
C29	0.062 (2)	0.042 (2)	0.0306 (17)	0.0027 (16)	0.0005 (14)	−0.0022 (14)

### Geometric parameters (Å, °)

**Table:**

O1—C14	1.358 (3)	C13—C14	1.392 (3)
O1—H1	0.8400	C13—H13	0.9500
O2—C18	1.235 (3)	C14—C15	1.381 (4)
O3—C25	1.252 (3)	C15—C16	1.383 (3)
N1—C1	1.385 (3)	C15—H15	0.9500
N1—C5	1.394 (3)	C16—H16	0.9500
N1—C6	1.492 (3)	C17—C25	1.434 (3)
C1—C2	1.367 (3)	C17—C18	1.454 (3)
C1—C9	1.461 (3)	C18—C19	1.499 (3)
C2—C3	1.407 (3)	C19—C20	1.374 (3)
C2—H2	0.9500	C19—C24	1.399 (3)
C3—C4	1.414 (3)	C20—C21	1.393 (4)
C3—C17	1.421 (3)	C20—H20	0.9500
C4—C5	1.376 (3)	C21—C22	1.369 (4)
C4—H4	0.9500	C21—H21	0.9500
C5—C26	1.539 (3)	C22—C23	1.391 (4)
C6—C7	1.533 (4)	C22—H22	0.9500
C6—H6A	0.9900	C23—C24	1.376 (3)
C6—H6B	0.9900	C23—H23	0.9500
C7—C8	1.515 (3)	C24—C25	1.494 (4)
C7—H7A	0.9900	C26—C27	1.531 (3)
C7—H7B	0.9900	C26—C28	1.539 (3)
C8—H8A	0.9800	C26—C29	1.540 (4)
C8—H8B	0.9800	C27—H27A	0.9800
C8—H8C	0.9800	C27—H27B	0.9800
C9—C10	1.337 (3)	C27—H27C	0.9800
C9—H9	0.9500	C28—H28A	0.9800
C10—C11	1.462 (3)	C28—H28B	0.9800
C10—H10	0.9500	C28—H28C	0.9800
C11—C16	1.395 (3)	C29—H29A	0.9800
C11—C12	1.396 (4)	C29—H29B	0.9800
C12—C13	1.376 (3)	C29—H29C	0.9800
C12—H12	0.9500		
			
C14—O1—H1	109.5	C14—C15—H15	120.1
C1—N1—C5	120.9 (2)	C16—C15—H15	120.1
C1—N1—C6	115.1 (2)	C15—C16—C11	121.6 (3)
C5—N1—C6	123.9 (2)	C15—C16—H16	119.2
C2—C1—N1	120.1 (2)	C11—C16—H16	119.2
C2—C1—C9	119.7 (2)	C3—C17—C25	126.5 (2)
N1—C1—C9	120.2 (2)	C3—C17—C18	125.6 (2)
C1—C2—C3	122.3 (2)	C25—C17—C18	108.0 (2)
C1—C2—H2	118.8	O2—C18—C17	129.4 (3)
C3—C2—H2	118.8	O2—C18—C19	123.4 (2)
C2—C3—C4	115.0 (2)	C17—C18—C19	107.2 (2)
C2—C3—C17	121.4 (2)	C20—C19—C24	121.3 (3)
C4—C3—C17	123.5 (2)	C20—C19—C18	130.1 (2)
C5—C4—C3	124.1 (2)	C24—C19—C18	108.5 (2)
C5—C4—H4	117.9	C19—C20—C21	117.9 (3)
C3—C4—H4	117.9	C19—C20—H20	121.0
C4—C5—N1	117.5 (2)	C21—C20—H20	121.0
C4—C5—C26	120.0 (2)	C22—C21—C20	120.9 (3)
N1—C5—C26	122.5 (2)	C22—C21—H21	119.5
N1—C6—C7	114.4 (2)	C20—C21—H21	119.5
N1—C6—H6A	108.7	C21—C22—C23	121.2 (3)
C7—C6—H6A	108.7	C21—C22—H22	119.4
N1—C6—H6B	108.7	C23—C22—H22	119.4
C7—C6—H6B	108.7	C24—C23—C22	118.3 (3)
H6A—C6—H6B	107.6	C24—C23—H23	120.9
C8—C7—C6	110.5 (2)	C22—C23—H23	120.9
C8—C7—H7A	109.5	C23—C24—C19	120.3 (3)
C6—C7—H7A	109.5	C23—C24—C25	131.7 (2)
C8—C7—H7B	109.5	C19—C24—C25	108.0 (2)
C6—C7—H7B	109.5	O3—C25—C17	128.0 (3)
H7A—C7—H7B	108.1	O3—C25—C24	123.6 (2)
C7—C8—H8A	109.5	C17—C25—C24	108.3 (2)
C7—C8—H8B	109.5	C27—C26—C28	105.0 (2)
H8A—C8—H8B	109.5	C27—C26—C5	110.5 (2)
C7—C8—H8C	109.5	C28—C26—C5	110.1 (2)
H8A—C8—H8C	109.5	C27—C26—C29	105.2 (2)
H8B—C8—H8C	109.5	C28—C26—C29	110.8 (2)
C10—C9—C1	123.2 (3)	C5—C26—C29	114.7 (2)
C10—C9—H9	118.4	C26—C27—H27A	109.5
C1—C9—H9	118.4	C26—C27—H27B	109.5
C9—C10—C11	127.0 (3)	H27A—C27—H27B	109.5
C9—C10—H10	116.5	C26—C27—H27C	109.5
C11—C10—H10	116.5	H27A—C27—H27C	109.5
C16—C11—C12	117.4 (2)	H27B—C27—H27C	109.5
C16—C11—C10	119.1 (3)	C26—C28—H28A	109.5
C12—C11—C10	123.4 (2)	C26—C28—H28B	109.5
C13—C12—C11	121.4 (2)	H28A—C28—H28B	109.5
C13—C12—H12	119.3	C26—C28—H28C	109.5
C11—C12—H12	119.3	H28A—C28—H28C	109.5
C12—C13—C14	120.1 (3)	H28B—C28—H28C	109.5
C12—C13—H13	120.0	C26—C29—H29A	109.5
C14—C13—H13	120.0	C26—C29—H29B	109.5
O1—C14—C15	122.8 (2)	H29A—C29—H29B	109.5
O1—C14—C13	117.6 (2)	C26—C29—H29C	109.5
C15—C14—C13	119.6 (2)	H29A—C29—H29C	109.5
C14—C15—C16	119.9 (3)	H29B—C29—H29C	109.5
			
C5—N1—C1—C2	0.8 (4)	C2—C3—C17—C18	−2.8 (4)
C6—N1—C1—C2	−175.6 (2)	C4—C3—C17—C18	178.4 (2)
C5—N1—C1—C9	−178.1 (2)	C3—C17—C18—O2	2.5 (5)
C6—N1—C1—C9	5.5 (3)	C25—C17—C18—O2	−179.3 (3)
N1—C1—C2—C3	2.0 (4)	C3—C17—C18—C19	−179.1 (2)
C9—C1—C2—C3	−179.1 (2)	C25—C17—C18—C19	−1.0 (3)
C1—C2—C3—C4	−3.8 (4)	O2—C18—C19—C20	0.6 (5)
C1—C2—C3—C17	177.3 (2)	C17—C18—C19—C20	−177.9 (3)
C2—C3—C4—C5	3.1 (4)	O2—C18—C19—C24	178.6 (3)
C17—C3—C4—C5	−178.1 (3)	C17—C18—C19—C24	0.1 (3)
C3—C4—C5—N1	−0.5 (4)	C24—C19—C20—C21	0.0 (4)
C3—C4—C5—C26	176.7 (2)	C18—C19—C20—C21	177.7 (3)
C1—N1—C5—C4	−1.5 (4)	C19—C20—C21—C22	−0.6 (4)
C6—N1—C5—C4	174.5 (2)	C20—C21—C22—C23	0.4 (4)
C1—N1—C5—C26	−178.7 (2)	C21—C22—C23—C24	0.3 (4)
C6—N1—C5—C26	−2.6 (4)	C22—C23—C24—C19	−0.9 (4)
C1—N1—C6—C7	−78.9 (3)	C22—C23—C24—C25	−178.6 (3)
C5—N1—C6—C7	104.9 (3)	C20—C19—C24—C23	0.8 (4)
N1—C6—C7—C8	153.0 (2)	C18—C19—C24—C23	−177.4 (2)
C2—C1—C9—C10	26.8 (4)	C20—C19—C24—C25	179.0 (2)
N1—C1—C9—C10	−154.3 (2)	C18—C19—C24—C25	0.8 (3)
C1—C9—C10—C11	−174.3 (2)	C3—C17—C25—O3	−1.5 (5)
C9—C10—C11—C16	−172.4 (3)	C18—C17—C25—O3	−179.7 (3)
C9—C10—C11—C12	8.6 (4)	C3—C17—C25—C24	179.6 (2)
C16—C11—C12—C13	0.1 (4)	C18—C17—C25—C24	1.5 (3)
C10—C11—C12—C13	179.1 (2)	C23—C24—C25—O3	−2.4 (5)
C11—C12—C13—C14	−0.7 (4)	C19—C24—C25—O3	179.7 (2)
C12—C13—C14—O1	−178.3 (2)	C23—C24—C25—C17	176.5 (3)
C12—C13—C14—C15	1.1 (4)	C19—C24—C25—C17	−1.4 (3)
O1—C14—C15—C16	178.5 (2)	C4—C5—C26—C27	13.2 (3)
C13—C14—C15—C16	−0.9 (4)	N1—C5—C26—C27	−169.7 (2)
C14—C15—C16—C11	0.3 (4)	C4—C5—C26—C28	−102.3 (3)
C12—C11—C16—C15	0.1 (4)	N1—C5—C26—C28	74.8 (3)
C10—C11—C16—C15	−178.9 (2)	C4—C5—C26—C29	131.8 (3)
C2—C3—C17—C25	179.3 (2)	N1—C5—C26—C29	−51.1 (3)
C4—C3—C17—C25	0.6 (4)		

### Hydrogen-bond geometry (Å, °)

**Table:**

*D*—H···*A*	*D*—H	H···*A*	*D*···*A*	*D*—H···*A*
O1—H1···O3^i^	0.84	1.79	2.626 (2)	177.
C2—H2···O2	0.95	2.24	2.927 (3)	128.
C4—H4···O3	0.95	2.29	2.966 (3)	127.
C15—H15···O3^i^	0.95	2.55	3.210 (3)	127.
C16—H16···O1^ii^	0.95	2.57	3.500 (3)	166.
C29—H29B···O2^iii^	0.98	2.59	3.515 (3)	158.

Symmetry codes: (i) *x*−1, −*y*+1/2, *z*−1/2; (ii) −*x*, *y*−1/2, −*z*−1/2; (iii) *x*, −*y*+1/2, *z*+1/2.
